# Clinicopathologic and prognostic value of *HER2* alterations in non-small cell lung cancer

**DOI:** 10.1186/s12931-026-03864-3

**Published:** 2026-08-10

**Authors:** Yicong Lin, Qianqian Xue, Bingxia Zhang, Yue Wang, Qiang Zheng, Yan Jin, Yuan Li

**Affiliations:** 1 Department of Pathology, Fudan University Shanghai Cancer Center, Shanghai, China; 2 Department of Oncology, Shanghai Medical College, Fudan University, Shanghai, China; 3 Institute of Pathology, Fudan University, Shanghai, China; 4 Department of Thoracic Surgery, Fudan University Shanghai Cancer Center, Shanghai, China

## Abstract

**Background:**

Human epidermal growth factor receptor 2 (*HER2*) alterations represent emerging therapeutic targets in non-small cell lung cancer (NSCLC). However, the clinicopathological and prognostic values of *HER2* mutations, amplification, and over expression remain poorly defined, leading to uncertainty in their prognostic and therapeutic implications.

**Methods:**

We conducted a retrospective cohort study involving 4438 patients who underwent surgical resection for lung lesions and next-generation sequencing (NGS) at Fudan University Shanghai Cancer Center (FUSCC) between January 2019 and May 2023. A total of 395 patients with *HER2* gene alterations were initially identified, of whom 33 with metastases from extrapulmonary tumors were excluded. The final cohort comprised 362 patients with primary NSCLC harboring *HER2* gene alterations, including pathogenic mutations (*n* = 230, 63.54%), variants of uncertain significance (VUS) (*n* = 104, 28.73%), and amplification (*n* = 28, 7.73%). We comprehensively compared clinicopathologic characteristics, molecular co-alteration landscapes, and HER2 protein expression across these subtypes to evaluate their prognostic impact on disease-free survival (DFS) and overall survival (OS). HER2 immunohistochemistry results were available for 221 patients, and protein-expression analyses were restricted to this subset.

**Results:**

*HER2* pathogenic mutations predominated in younger, female, never-smokers with lung adenocarcinoma. In contrast, *HER2* amplification was associated with older age, male sex, smoking history, non-adenocarcinoma histology, and advanced pathological stage. Genomic profiling identified a distinct co-mutation landscape: *HER2* amplification harbored a significantly higher burden of concurrent *TP53* (82.1% vs. 20.0%) and *EGFR* (46.4% vs. 7.4%) mutations compared to pathogenic mutations. Among patients with available HER2 immunohistochemistry results, amplification was associated with higher HER2 protein expression, with 40.0% of cases showing an IHC score of 3+, whereas pathogenic mutations and VUS were predominantly HER2-negative (IHC 0 in 77.3% and 81.7%, respectively; *P* < 0.001). *HER2* amplification was associated with inferior DFS and OS in Kaplan–Meier analyses and with shorter DFS in univariable Cox analysis; however, it was not retained as an independent prognostic factor after adjustment for clinicopathological variables or postoperative adjuvant treatment. The Y772_A775dup insertion mutation showed relatively favorable survival outcomes in exploratory mutation-subgroup analyses.

**Conclusions:**

*HER2* alterations in surgically resected NSCLC are associated with heterogeneous clinicopathological, genomic, protein-expression, and survival profiles. *HER2* amplification was associated with adverse clinicopathological features and inferior unadjusted survival, but its association with DFS was not independent of clinicopathological factors and postoperative treatment. The favorable survival findings associated with Y772_A775dup should be considered exploratory. These results support alteration-specific interpretation of *HER2* findings and further validation in larger, treatment-annotated cohorts.

**Supplementary Information:**

The online version contains supplementary material available at https://doi.org/10.1186/s12931-026-03864-3.

## Introduction

 Lung cancer remains one of the leading causes of mortality worldwide. Non–small cell lung cancer (NSCLC), which accounts for 85% of all lung cancer cases [1], represents a heterogeneous group of diseases driven by diverse genomic alterations [2]. The integration of comprehensive molecular profiling, including next-generation sequencing (NGS), has become essential for guiding diagnosis and treatment decisions in the era of precision oncology [3].

The human epidermal growth factor receptor 2 (*HER2*, also known as *ERBB2*) is a member of the *ERBB* receptor tyrosine kinase family that regulates key cellular processes such as proliferation, differentiation, and survival [4]. While *HER2* amplification and overexpression have been well documented to play a role in the development of breast cancer and gastric cancer [5, 6], their biological and clinical implications in NSCLC remain incompletely understood. *HER2* alterations, including mutations, amplification, and protein overexpression, and these alterations do not necessarily overlap [7, 8]. The prevalence of these subtypes varies significantly. Among patients with NSCLC, *HER2* mutations occur in approximately 2–4% of cases [9], amplification occurs in approximately 3%, and protein overexpression is reported at widely variable rates (2.4%–38%)^7^. Given this heterogeneity, it is necessary to define the optimal diagnostic strategy for *HER2* alterations in NSCLC. *HER2* alterations can be broadly categorized into three main groups based on genomic profiling by NGS: pathogenic mutations, amplification, and variants of uncertain significance (VUS). Pathogenic mutations, primarily in-frame insertions in exon 20 of the *HER2* gene, account for over 50% of all *HER2* alterations and are found in 1.5–1.7% of NSCLC patients [10, 11]. These mutations have been identified as oncogenic drivers and actionable therapeutic targets, with antibody–drug conjugates (ADCs) showing promising efficacy [12]. Conversely, *HER2* amplification and protein overexpression, traditionally assessed by fluorescence in situ hybridization (FISH) and immunohistochemistry (IHC), respectively, and may represent partially overlapping but biologically and clinically distinct manifestations of *HER2* dysregulation [8]. Furthermore, the increasing use of NGS has led to the frequent detection of *HER2* VUS, which comprise a biologically heterogeneous category of variants with largely undefined functional, clinicopathological, and prognostic implications, posing significant clinical challenges [13].

Previous studies have explored the correlation between *HER2* amplification (detected by FISH) and HER2 overexpression (detected by IHC) in lung cancer have shown an unreliable correlation and widely variable positivity rates [14–16]. Additionally, the association between *HER2* gene alterations (detected by NGS) and HER2 protein expression (detected by IHC) remains unclear. A better understanding of this relationship is crucial to clarify the molecular heterogeneity of *HER2* gene-altered NSCLC and improve diagnostic stratification in clinical practice.

In addition to these diagnostic challenges, emerging evidence suggests that different *HER2* gene alteration subtypes may carry distinct prognostic implications [17, 18]. Previous studies have reported that *HER2* gene-altered NSCLC was associated with poor survival outcomes compared with wild-type cases [19]. However, survival heterogeneity across *HER2* pathogenic mutations, amplification, and the heterogeneous VUS category has not been systematically evaluated. Therefore, in this retrospective cohort of surgically resected NSCLC, we conducted a comprehensive analysis of the clinicopathological characteristics, genomic co-mutation landscapes, HER2 protein expression, and prognostic outcomes across distinct *HER2* gene alteration subtypes. This study aimed to characterize the heterogeneity of *HER2* alterations in surgically resected NSCLC and to provide a basis for further clinical and biological investigation.

## Materials and methods

### Patient collection

We retrospectively screened patients who underwent surgical resection for lung lesions and NGS at Fudan University Shanghai Cancer Center (FUSCC) between January 1, 2019 to May 31, 2023. Patients with pathologically confirmed NSCLC harboring *HER2* gene alterations were included in this study. Patients whose resected lung lesions were postoperatively confirmed as metastases from extrapulmonary primary tumors were excluded. The clinical data and molecular results were obtained from the patients’ electronic medical records. Clinicopathological parameters including age, gender, smoking history, histology, pathological stage, pathological subtype, and features such as vascular invasion (VI), perineural invasion (PNI) and pleural invasion (PI) were recorded. The follow-up of this patient cohort was completed on August 1, 2025, and the median length of follow-up was 41.2 months. Overall survival (OS) was defined as the time from the initial diagnosis until death from any cause. Disease-free survival (DFS) was defined as the period from surgery to recurrence or the last follow-up. Patients without events were censored from the time point of the last follow-up. The study was approved by the Ethics Committee of the FUSCC (IRB No. 050432-4-2108*) and was conducted in accordance with the Declaration of Helsinki. Written informed consent was obtained from all participants.

### Next-generation sequencing testing

Genomic DNA was extracted from formalin fixed and paraffin embedded (FFPE) tissue samples using the QIAamp DNA Mini Kit. Targeted deep sequencing of mutational hotspots was conducted using a capture-based targeted sequencing panel. *HER2* gene status, including pathogenic mutations, amplification and VUS, was assessed using two NGS panels based on the Illumina NextSeq: 68-gene or 520-gene panels (Burning Rock Dx, Guangzhou, China), which are routinely used at FUSCC to detect genomic alterations. Sequencing was performed according to the manufacturer’s instructions. *HER2* amplification was classified according to the clinical NGS report as *ERBB2* copy-number amplification. *ERBB2* copy number was estimated from normalized sequencing depth across targeted *ERBB2* regions. According to the reporting criteria used by the clinical NGS platform, an estimated *ERBB2* copy number of ≥ 4 was generally considered indicative of copy-number amplification, while the final classification also incorporated the overall copy-number background of the sample and assay quality-control metrics. Only cases explicitly reported as *ERBB2* amplification in the clinical sequencing report were assigned to the *HER2* amplification subgroup.

### Immunohistochemistry

Tissues from surgical resections were FFPE for the assessment of both HER2 (4B5) and programmed death-ligand 1 (PD-L1) status by IHC. In this retrospective cohort, HER2 IHC was not routinely performed for all patients during clinical management and was not conducted according to a uniform prospective testing protocol. Instead, HER2 IHC testing was performed according to routine clinical practice during the study period. Therefore, analyses involving HER2 protein expression were restricted to patients with available HER2 IHC results. Staining was performed on a Ventana BenchMark Ultra autostainer (Ventana Medical System Inc, Roche, Tucson, Arizona) following the manufacturer’s standardized protocol [20]. HER2 expression was evaluated based on the percentage of positive tumor cells, staining intensity, and membrane staining pattern (complete, incomplete, or basolateral/lateral), in accordance with the modified 2017 American Society of Clinical Oncology (ASCO)/College of American Pathologists (CAP) HER2 testing guidelines for gastric cancer [21]. PD-L1 IHC testing was performed using the 22C3 antibody clone (DAKO) as previously described in detail [22]. The tumor proportion score (TPS) was defined as the percentage of tumor cells with complete or partial membranous staining at any intensity. PD-L1 expression was categorized into three groups: negative (TPS < 1%), low expression (TPS 1–49%), and high expression (TPS ≥ 50%). All IHC stained sections were independently reviewed by two pathologists (Y.Li and Y.J.) who were blinded to the clinical information.

### Statistical analysis

Associations between *HER2* alterations and clinicopathological characteristics were assessed using the chi-square test or Fisher’s exact test as appropriate. Univariate analysis was performed using Cox proportional hazards regression model, which included potential confounders such as age, sex, histology, histological differentiation, pathological stage, VI, PI, PNI, PD-L1 expression and *HER2* status. Variables with a *P* value of < 0.05 in the univariate analysis were included in the multivariate Cox regression model. Survival outcomes (DFS and OS) were performed by Kaplan–Meier curves, and differences were evaluated with the log-rank test. A two-sided *P* value of < 0.05 was considered statistically significant. All analyses were performed using R studio (version 3.4.2) and GraphPad Prism 8.

## Results

### Comparison of clinicopathologic features among heterogeneous *HER2* gene alterations

**Fig. 1:**
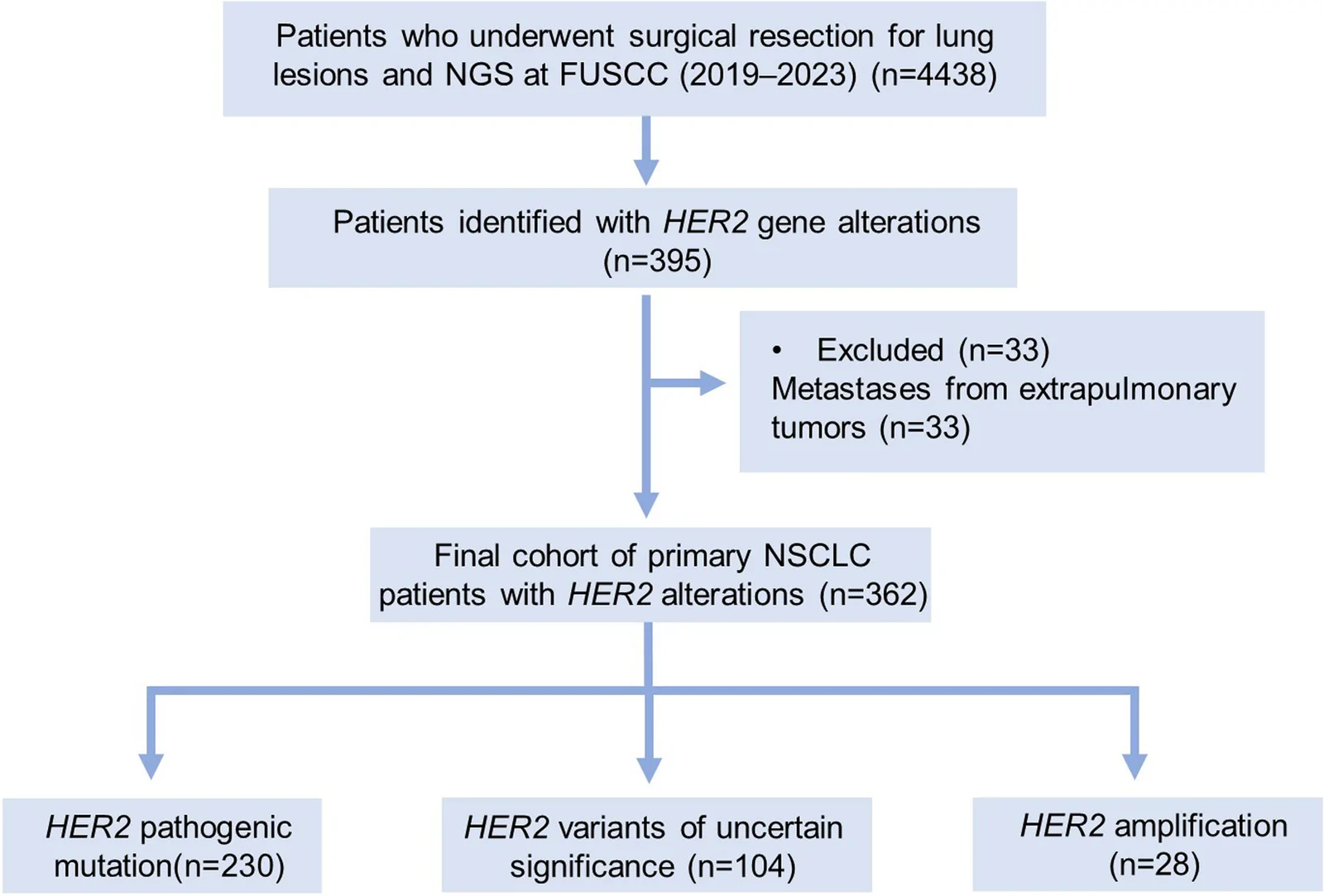
Flow chart of patient selection process. *HER2* pathogenic mutation (*n* = 230), *HER2* variants of uncertain significance (VUS) (*n* = 104), and *HER2* amplification (*n* = 28). NGS, Next-generation sequencing; FUSCC, Fudan University Shanghai Cancer Center; *HER2*, Human epidermal growth factor receptor 2; NSCLC, Non-small cell lung cancer

A total of 4,438 patients who underwent surgical resection for lung lesions and NGS at FUSCC during the study period were assessed. Of these, 395 patients were identified as harboring *HER2* gene alterations. After excluding 33 patients whose lung lesions were confirmed postoperatively as metastases from extrapulmonary tumors, 362 patients were included in the final cohort. These patients were categorized into three groups based on the type of *HER2* gene alteration: 230 patients with pathogenic mutations, 104 with VUS, and 28 with amplification (Fig. 1). The clinicopathological characteristics of the cohort are summarized in Table 1, and several characteristics differed significantly across the three *HER2* alteration categories. Patients with *HER2* pathogenic mutation category were younger at diagnosis compared to those with amplification and VUS categories (*P* < 0.0001). The pathogenic mutation category also exhibited a significantly higher proportion of female patients (68.70%) than the amplification (35.71%) and VUS (42.31%) groups (*P* < 0.0001). Furthermore, the pathogenic mutation category included a significantly higher percentage of never-smokers (83.04%) relative to the amplification (42.86%) and VUS (55.77%) categories (*P* < 0.0001). Histologically, the majority of *HER2* mutation cases were lung adenocarcinoma (LUAD) (228/230, 99.13%), whereas 75.00% (21/28) of amplification patients had LUAD, 14.29% (4/28) had lung squamous cell carcinoma (LUSC), and 10.71% (3/28) had other histological types. Notably, compared with the *HER2* mutation and VUS categories, the *HER2* amplification group was associated with a poorly-differentiation (*P* < 0.0001), more advanced pathologic T stages (*P* = 0.0031) and pathologic N stages (*P* = 0.0375), advanced pathological stage (*P* = 0.0003), and increased VI incidence (*P* < 0.0001). The detailed distributions of pT stage, pN stage, and pathological stage for all *HER2*-altered NSCLC patients are provided in Supplementary Table 1. We further stratified the alterations into subgroups based on specific *HER2* alterations, including Y772_A775dup, G776delinsVC, S310F, V659E, G778_P780dup, amplification combined with mutation, other pathogenic mutations, VUS, and amplification. The Y772_A775dup was the most frequent pathogenic mutation subtype (141/230, 61.30%). Significant clinicopathological heterogeneity exists among *HER2* gene alteration categories, which show distinct associations with age, sex, smoking history, histology, histological differentiation, pathological stage, and the presence of VI (Supplementary Table 2). Overall, these findings demonstrate clinicopathological differences across *HER2* alteration categories in this surgically resected NSCLC cohort. However, because *HER2* VUS represents a heterogeneous category of variants with uncertain functional significance, findings related to the VUS category should be considered descriptive and exploratory rather than interpreted as evidence of a biologically homogeneous subgroup.

**Table 1:** Clinicopathologic characteristics of patients with *HER2* gene-altered NSCLC

	Number of patients (%) with HER2 alteration			
	Mutation		Amplification	
Variables	Pathogenic mut (*n* = 230)	VUS (*n* = 104)	Amp (*n* = 28)	*p* value
Age				< 0.0001
* ≤*59 years	165(71.74)	36(34.62)	7(25.00)	
>59 years	65(28.26)	68(65.38)	21(75.00)	
Sex				< 0.0001
Female	158(68.70)	44(42.31)	10(35.71)	
Male	72(31.30)	60(57.69)	18(64.29)	
Smoking				< 0.0001
Never	191(83.04)	58(55.77)	12(42.86)	
Ever	39(16.96)	46(44.23)	16(57.14)	
Histology				< 0.0001
LUAD	228(99.13)	86(82.69)	21(75.00)	
LUSC	0(0.00)	9(8.65)	4(14.29)	
Other	2(0.87)	9(8.65)	3(10.71)	
Differentiation				< 0.0001
Well-differentiated	37(16.09)	5(4.81)	0(0.00)	
Moderately-differentiated	69(30.00)	40(38.46)	2(7.14)	
Poorly-differentiated	51(22.17)	48(46.15)	25(89.29)	
NA	73(31.74)	11(10.58)	1(3.57)	
pT stage				0.0031
T1-T2	223(96.96)	95(91.35)	23(82.14)	
T3-T4	7(3.04)	9(8.65)	5(17.86)	
pN stage				0.0375
N0	199(86.52)	91(87.50)	19(67.86)	
N1-2	31(13.48)	13(12.50)	9(32.14)	
pStage				0.0003
I-II	205(89.13)	95(91.35)	17(60.71)	
III-IV	25(10.87)	9(8.65)	11(39.29)	
Vascular invasion				< 0.0001
Absent	181(78.70)	76(73.08)	10(35.71)	
Present	37(16.09)	22(21.15)	16(57.14)	
NA	12(5.22)	6(5.77)	2(7.14)	
Perineural invasion				0.1019
Absent	224(97.39)	98(94.23)	25(89.29)	
Present	4(1.74)	3(2.88)	2(7.14)	
NA	2(0.87)	3(2.88)	1(3.57)	
Pleural invasion				0.1775
Absent	194(84.35)	87(83.65)	19(67.86)	
Present	22(9.57)	12(11.54)	7(25.00)	
NA	14(6.09)	5(4.81)	2(7.14)	

*HER2* Human epidermal growth factor receptor 2, *Pathogenic mut* Pathogenic mutation, *VUS* Variants of uncertain significance, *Amp* Amplification, *pT stage* pathologic T stage, *pN stage* pathologic N stage, *pStage* pathological stage, *NA* Not available

### Genetic landscape of *HER2* gene-altered NSCLC

**Fig. 2:**
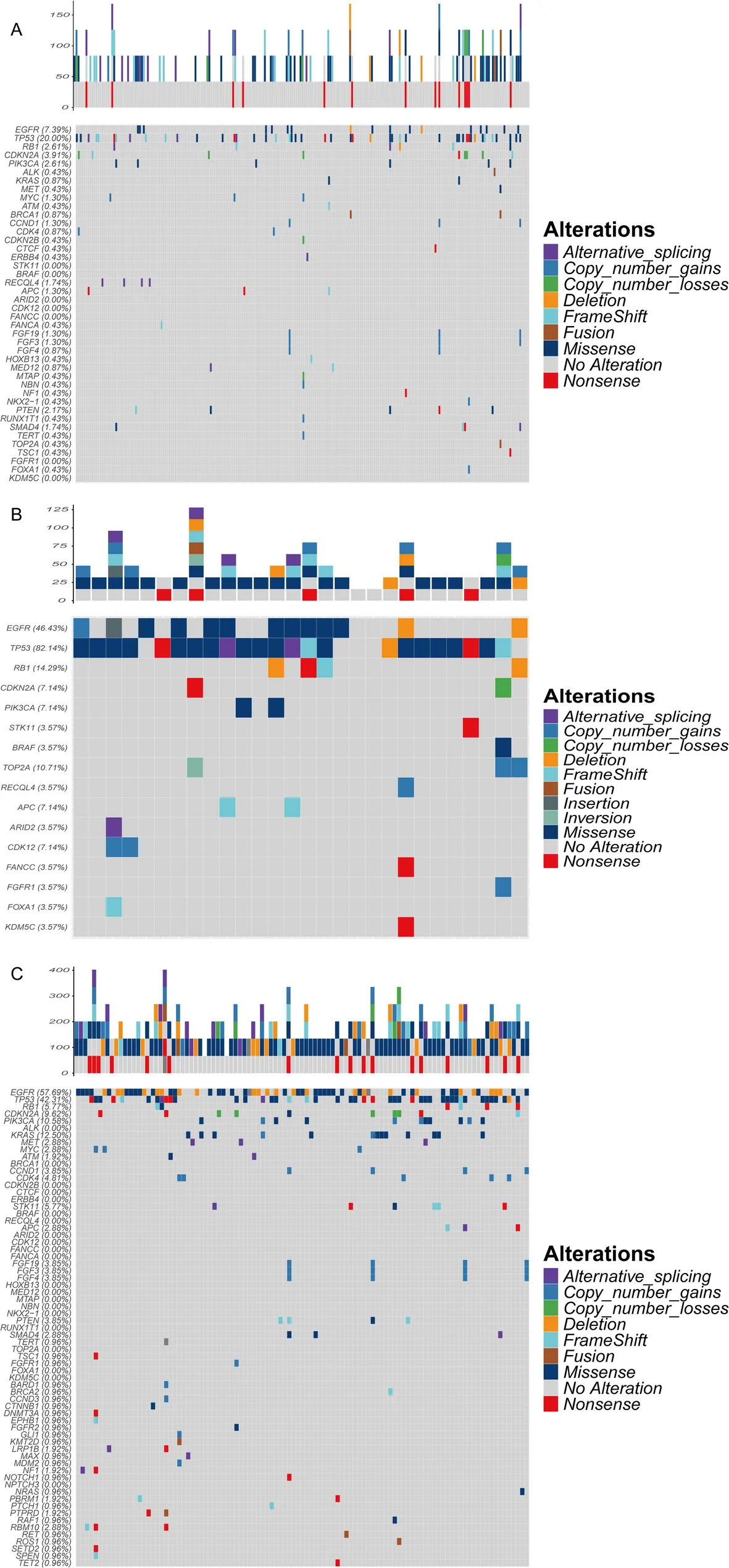
The genomic landscape of *HER2* gene-altered NSCLC, 2**A** (pathogenic mutations), 2**B** (amplification), and 2**C** (variants of uncertain significance)

To explore the landscape of co-occurring genomic alterations, we analyzed 362 *HER2* gene-altered NSCLC cases, revealing significant molecular heterogeneity across categories (Fig. 2). The *HER2* pathogenic mutation category showed relatively lower frequencies of *TP53* (20.00%) and *EGFR* (7.39%) co-alterations compared with the other categories (Fig. 2A). In contrast, the *HER2* amplification was associated with a higher frequency of co-occurring genomic alterations, including *TP53* co-mutations in 82.14% of cases and concurrent *EGFR* mutations in 46.43% of cases (Fig. 2B). The *HER2* VUS category exhibited a different distribution of co-occurring alterations, characterized by a predominance of *EGFR* mutations (57.69%), which surpassed *TP53* in frequency (42.31%). Other recurrent alterations in the VUS category included *KRAS* (12.5%), *PIK3CA* (10.58%), *CDKN2A* (9.62%) and so on (Fig. 2C). Given the uncertain functional significance of VUS, these findings should be interpreted as descriptive and exploratory. Collectively, these results demonstrate molecular heterogeneity among NSCLC tumors harboring different categories of *HER2* alterations. *HER2* amplification was associated with a more complex co-alteration pattern, particularly involving *TP53* and *EGFR*, whereas *HER2* pathogenic mutations showed fewer selected co-alterations in this cohort. However, the biological significance of the VUS category requires cautious interpretation and further validation.

### HER2 immunoreactivity heterogeneity and genomic associations in *HER2* gene-altered NSCLC

**Fig. 3:**
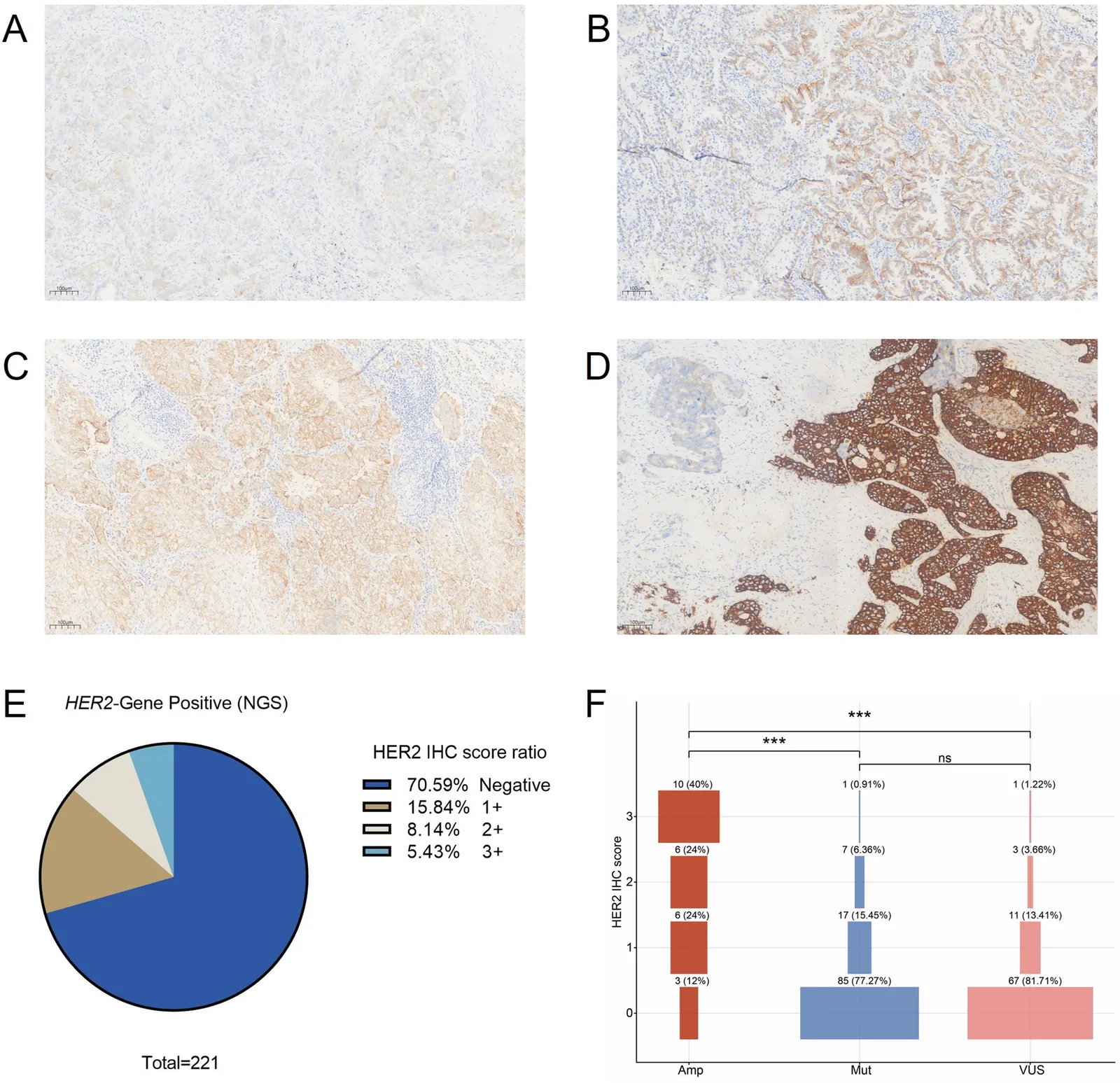
Correlation of HER2 status assessment across protein expression level and genomic alteration status. **A** 1+: tumor cells with faint/barely perceptible membranous staining. **B** 2+ (pattern 1): tumor cells with weak to moderate complete, basolateral and lateral membranous staining. Columnar cells that are sectioned tangentially tend to show a complete membranous staining pattern. **C** 2+(pattern 2): tumor cells with weak to moderate complete membranous staining. **D** 3+: tumor cells with a strong complete, basolateral and lateral membranous reactivity. Scale bars = 100 μm. **E** The distribution of IHC scores in this subset of HER2-gene alteration cases. **F** The relationship between HER2 IHC score and HER2-gene alteration category (Four cases with concurrent pathogenic HER2 mutation and HER2 amplification were excluded from the category-specific comparison). ns, not significant; ***, *P* < 0.001

HER2 protein expression assessed by IHC showed a spectrum of staining patterns and intensities, which were scored according to the modified ASCO/CAP guidelines for gastric cancer [21]. Representative IHC images of NSCLC specimens are shown in Fig. 3A and D. Specifically, HER2 (1+) was assigned to tumors with faint or barely perceptible, incomplete membrane staining observed in ≥ 10% of tumor cells (Fig. 3A). HER2 (2+) was assigned to tumors with weak to moderate membrane staining in ≥ 10% of cells, which could present in two distinct patterns. The first pattern was localized to the basolateral or lateral aspects of the tumor cell membrane (Fig. 3B). The other pattern was the staining encompassed the entire circumference of the tumor cell membrane (Fig. 3C). Lastly, HER2 (3+) was characterized by strong basolateral, lateral, or complete circumferential membrane staining in ≥ 10% of tumor cells. Notably, intratumoral heterogeneity (ITH) of HER2 expression was observed in a subset of cases. These specimens displayed distinct regional variations in staining intensity, such as the coexistence of strongly positive (3+) clusters adjacent to negative or weakly stained areas within the same tissue section (Fig. 3D).

Among the 362 patients with *HER2*-altered NSCLC, 221 had available HER2 IHC results. Therefore, analyses involving HER2 protein expression were restricted to patients with available HER2 IHC data. The distribution of IHC scores in this subset of *HER2* gene-altered cases was as follows: 70.58% (156/221) of patients exhibited negative HER2 expression (IHC 0), 15.83% (35/221) exhibited HER2 1+, 8.14% (18/221) showed HER2 2+, and 5.43% (12/221) demonstrated HER2 3+ (Fig. 3E).

We further explored the association between HER2 protein expression and *HER2* gene alteration category in patients with available IHC results (Fig. 3F). The *HER2* amplification group (*n* = 25) was strongly associated with high protein expression, with 40% of patients (10/25) displaying 3 + membranous staining, and 48% (12/25) showing a 1 + to 2 + pattern. In contrast, the *HER2* mutation subgroup (*n* = 110) was characterized by predominantly absent HER2 protein expression: 77.27% (*n* = 85) of cases had an IHC score of 0, with intermediate IHC scores (1 and 2) observed in 15.45% (*n* = 17) and 6.36% (*n* = 7) of patients, respectively; only 0.91% (*n* = 1) achieved an IHC score of 3. A similar distribution was observed in the *HER2* VUS category (*n* = 82): 81.71% (*n* = 67) had IHC 0, 13.41% (*n* = 11) had IHC 1+, 3.66% (*n* = 3) had IHC 2+, and 1.22% (*n* = 1) had a IHC 3+. Statistically, the HER2 IHC score distribution differed significantly between the amplification and pathogenic mutation categories (*P* < 0.001), whereas no significant difference was detected between the pathogenic mutation and VUS categories. These findings suggest an association between *HER2* alteration category and HER2 protein expression among patients with available IHC results. *HER2* amplification was associated with a higher frequency of strong membranous staining, whereas *HER2* pathogenic mutations and VUS were more frequently associated with absent or low-level HER2 protein expression.

### Association of *HER2* gene alterations with histological subtypes and PD-L1 expression

**Fig. 4:**
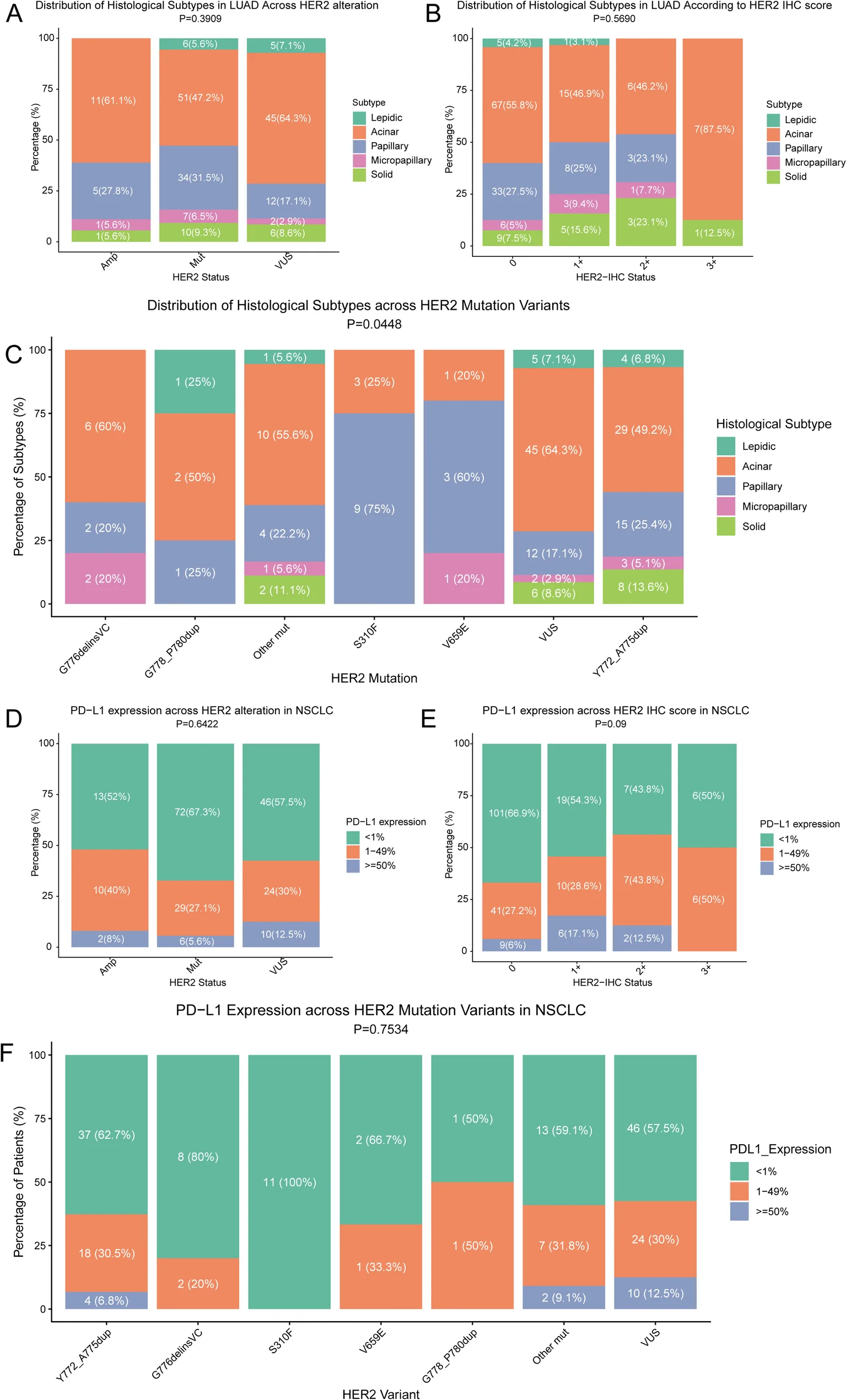
Association of *HER2* gene alterations with histological subtypes and PD-L1 expression. **A**-**B** The distribution of histological subtypes in LUAD according to *HER2* gene alteration types and protein expression level. **C** Variation in histological patterns among specific *HER2* mutation variants. **D**-**E** The distribution of PD-L1 expression in NSCLC according to *HER2* gene alteration types and protein expression level. **F** PD-L1 expression status across specific *HER2* mutation variants

To further investigate the phenotype of *HER2* gene-altered NSCLC, we examined the relationship between *HER2* genomic and protein status and clinicopathological features, including LUAD histological subtypes and PD-L1 expression. Across the entire cohort, the acinar subtype was the predominant histological pattern. Stratification by *HER2* alterations (amplification, pathogenic mutation, and VUS; Fig. 4A) or by HER2 protein expression level (IHC 0–3+; Fig. 4B) revealed no significant differences in histological distribution (*P* = 0.3909 and *P* = 0.5690, respectively). However, differences in histological patterns were observed when analyzing specific *HER2* variants (*P* = 0.0448; Fig. 4C). Notably, the papillary subtype appeared more frequent in tumors harboring S310F (75%) and V659E (60%) variants than in those harboring Y772_A775dup (25.4%) or G776delinsVC (20%), although sample sizes for some rare variants were limited. Conversely, the acinar subtype remained predominant in VUS (64.3%) and the Y772_A775dup (49.2%) groups.

Regarding immune-related features, PD-L1 expression was generally low and showed no significant association with *HER2* status. The distribution of PD-L1 TPS was comparable across *HER2* gene alteration categories (*P* = 0.6422; Fig. 4D), with the majority of tumors being PD-L1 negative (TPS < 1%). Although higher HER2 IHC scores showed a trend toward increased PD-L1 positivity, this association did not reach statistical significance (*P* = 0.09; Fig. 4E). Similarly, no significant differences in PD-L1 expression patterns were observed among specific *HER2* variant groups (*P* = 0.7534; Fig. 4F). Of note, tumors harboring the S310F mutation were uniformly PD-L1 negative (11/11), whereas high PD-L1 expression (TPS ≥ 50%) was sporadically observed in the VUS (12.5%) and Y772_A775dup (6.8%) groups. Taken together, these results indicate that broad *HER2* alteration categories and HER2 protein expression levels were not significantly associated with LUAD histological subtype, whereas some variant-level differences in histological patterns were observed. *HER2* gene-altered NSCLC was generally characterized by low PD-L1 expression in this cohort, suggesting a relatively low PD-L1 immune profile.

### The prognostic value of *HER2* alterations in NSCLC

**Fig. 5:**
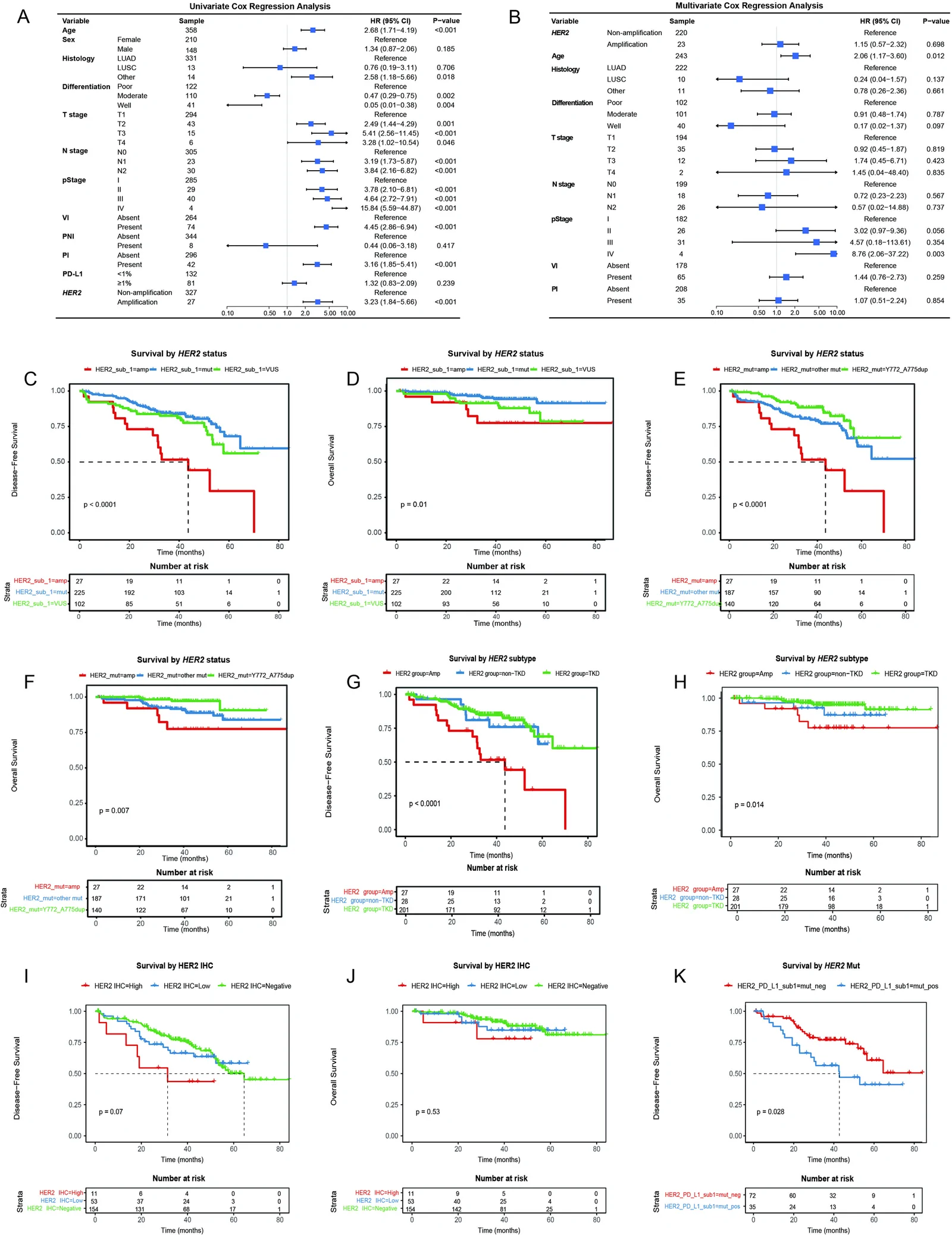
The prognostic value of *HER2* alterations in NSCLC. **A**-**B** Univariate Cox analysis and multivariate Cox analyses were performed to assess the impact of various clinical and pathologic factors on DFS. **C**-**D** Kaplan-Meier plot for DFS/OS in all patients harboring *HER2* alterations. **E**-**F** Kaplan-Meier plot for DFS/OS of the *HER2* amplification vs. other mutation vs. Y772_A775dup. **G**-**H** Kaplan-Meier plot for DFS/OS of the *HER2* TKD vs. non-TKD vs. amplification. **I**-**J** Kaplan-Meier plot for DFS/OS of the HER2 strong protein expression (3+) vs. the lower group (1 + and 2+) vs. negative group. (K) Kaplan-Meier plot for DFS of the *HER2* mutation (PD-L1^+^) vs. the *HER2* mutation (PD-L1^−^)

Univariate Cox regression analysis identified older age, advanced pT and pN stage, higher pStage, the presence of VI and PI, and *HER2* amplification as factors associated with shorter DFS in patients with *HER2*-altered NSCLC (Fig. 5A). After incorporating these variables into a multivariable Cox model, older age and advanced pStage remained independent predictors of shorter DFS, whereas *HER2* amplification was not retained as an independent prognostic factor (Fig. 5B).

Kaplan-Meier survival analysis showed heterogeneous survival outcomes according to *HER2* genomic and protein status. Patients harboring *HER2* amplification had inferior outcomes compared with those with pathogenic mutations or VUS, both in terms of DFS (*P* < 0.0001; Fig. 5C) and OS (*P* = 0.01; Fig. 5D). Further stratification of the mutation cohort revealed that patients harboring the Y772_A775dup insertion mutation (the most prevalent subtype) had a notably better prognosis than those with *HER2* amplification and showed a trend toward favorable outcomes compared with other mutation types (DFS, *P* < 0.001, Fig. 5E; OS, *P* = 0.007, Fig. 5F). When *HER2* mutations were further categorized by tyrosine kinase domain (TKD) status, patients with TKD mutations showed the most favorable outcomes. In contrast, the *HER2* amplification group exhibited the shortest DFS and OS, while patients with non-TKD mutations displayed an intermediate prognosis, particularly in terms of OS (DFS, *P* < 0.0001, Fig. 5G; OS, *P* = 0.014, Fig. 5H). In contrast to *HER2* genomic profiling, HER2 protein expression failed to significantly stratify survival outcomes (Fig. 5I, J). Although a trend toward shorter DFS was observed in the HER2-High group (*P* = 0.07), this did not translate into a significant OS difference (*P* = 0.53), likely due to the limited sample size of the high HER2 expression subgroup (*n* = 11). Additionally, within the *HER2*-mutated population, PD-L1 positivity was associated with shorter DFS (*P* = 0.028, Fig. 5K). Available postoperative and post-recurrence treatment information was summarized in Supplementary Table 3.1 and 3.2. In an additional Cox analysis incorporating postoperative adjuvant treatment, *HER2* amplification was not retained as an independent predictor of DFS (HR = 1.17, 95% CI: 0.62–2.22, *P* = 0.631; Supplementary Table 3.3). This result was consistent in a sensitivity analysis restricted to patients with available postoperative treatment information (HR = 1.27, 95% CI: 0.66–2.45, *P* = 0.476). Collectively, *HER2* amplification was associated with inferior DFS and OS in Kaplan–Meier analyses and with shorter DFS in univariable Cox analysis, but was not retained as an independent prognostic factor in the multivariable or treatment-adjusted analyses. 

## Discussion

Understanding the molecular diversity of *HER2* gene alterations in NSCLC is critical for optimizing personalized treatment strategies [23]. This study provides a comprehensive characterization of *HER2* heterogeneity in a surgically resected NSCLC cohort, including the clinicopathological, genomic, IHC, and prognostic profiles associated with different *HER2* alteration categories. These findings highlight the heterogeneity of *HER2-*altered NSCLC and support further investigation of the clinical relevance of different *HER2* alteration categories.

Clinicopathological differences were observed across the *HER2* alteration categories. The *HER2* amplification cohort was predominantly male, exhibited a range of histological types, and frequently harbored concurrent driver mutations. In contrast, *HER2* pathogenic mutations were more frequently observed in younger, female, non-smoking patients, consistent with previous studies [9]. Patients in the VUS category were more likely to be older and male and to have poorly differentiated tumors in this cohort. However, *HER2* VUS represents a heterogeneous group of variants with uncertain and potentially variable functional significance. Therefore, these clinicopathological observations should be considered descriptive and exploratory and should not be interpreted as evidence that the VUS category represents a biologically homogeneous or uniformly aggressive subgroup.

The use of NGS has enabled a more comprehensive and detailed characterization of the genomic landscape in *HER2* gene-altered NSCLC [24]. Our study confirmed significant molecular heterogeneity across the different *HER2* alteration categories. The *HER2* amplification showed higher frequencies of selected co-occurring alterations, particularly in *TP53* (82.14%) and *EGFR* (46.43%). This aligns with observations in breast cancer, where *HER2* amplification is well recognized as a hallmark of genomic instability [25]. In NSCLC, these findings may reflect a more complex genomic context associated with *HER2* amplification, potentially including amplification arising in tumors driven by other oncogenic alterations. In contrast, pathogenic mutations were associated with lower frequencies of these selected co-occurring alterations, including *TP53* (20.0%) and *EGFR* (7.39%), supporting the notion that *HER2* pathogenic mutations function as independent oncogenic drivers, often representing early events in lung carcinogenesis [26], although the underlying biological mechanisms require further validation. Furthermore, the VUS group exhibited a different co-alteration pattern, characterized by a high frequency of *EGFR* co-mutations (57.69%), suggesting that many *HER2* VUS may be passenger events in tumors primarily driven by *EGFR* mutations. However, because *HER2* VUS represents a functionally heterogeneous category, the biological and clinical significance of individual variants remains uncertain. Therefore, these findings should be considered descriptive and exploratory and should not be interpreted as evidence of a uniform biological phenotype or as a basis for treatment selection.

Importantly, our study revealed a significant discordance between *HER2* gene alterations and protein expression. Specifically, 77.27% of patients with *HER2* pathogenic mutations exhibited negative protein expression (IHC 0), emphasizing that IHC alone is an unreliable tool for identifying *HER2* mutations. This limitation highlights the risk of excluding patients who could benefit from *HER2*-targeted therapies, such as *HER2*-directed ADCs, when relying solely on IHC for screening. Similar findings have been reported in breast cancer, where *HER2* mutations are often associated with HER2-negative or HER2-low tumors [27]. Conversely, high protein expression (IHC 3+) was strongly associated with *HER2* amplification, detected in 40% of the amplification cohort. These findings align with previous reports that have observed a correlation between IHC 3 + staining and *HER2* amplification in NSCLC, though HER2 IHC negative or low cases have also been found to be FISH-positive [28]. An additional important observation in this study was the increased rate of strong HER2 positivity (IHC 3+) within the subset of *HER2* gene-altered NSCLC patients identified by NGS. While HER2 overexpression is generally low in NSCLC [29], the frequency of strong HER2 expression (IHC 3+) was notably higher in the NGS *HER2*-positive cohort, reinforcing the importance of NGS in identifying patients who may benefit from *HER2*-targeted therapies. Moreover, it is essential to consider HER2 ITH, which could contribute to the observed discordance between *HER2* gene alterations and protein expression. HER2 ITH within a tumor may affect the expression of the protein in different tumor regions, potentially leading to underrepresentation of *HER2* alterations. Furthermore, this ITH of HER2 expression can impact therapeutic response, as has been previously suggested for breast cancers with HER2 overexpression [27, 30]. These data highlight the necessity of comprehensive genomic testing in detection of *HER2* gene-altered NSCLC, allowing implementation of optimal therapeutic strategies [31].

We observed heterogeneous survival outcomes across the different *HER2* gene alteration categories. *HER2* amplification was associated with inferior DFS and OS in Kaplan–Meier analyses and with shorter DFS in univariable Cox analysis, but it was not retained as an independent prognostic factor after adjustment for clinicopathological variables and postoperative adjuvant treatment. The adverse survival pattern in the amplification category may therefore have been influenced by more advanced disease and treatment-related imbalance. The association between postoperative adjuvant treatment and shorter DFS likely reflects confounding by indication rather than an adverse treatment effect. Only one patient received trastuzumab deruxtecan after recurrence or metastasis, precluding a treatment-specific survival analysis. Conversely, Y772_A775dup was associated with the most favorable prognosis. Furthermore, we found that this survival benefit was not limited to the Y772_A775dup variant but extended to the entire class of TKD mutations. Our structural stratification revealed a clear prognostic gradient: patients with TKD mutations exhibited significantly better outcomes compared to those with non-TKD mutations or amplification. This finding significantly substantiates the trend recently reported by Lee Y, et al. [19]; which suggested a potential survival benefit for the TKD subgroup, our analysis provides the statistical confirmation that was previously lacking, firmly establishing this structural distinction as a significant prognostic determinant. The biological indolence of the TKD subtype is coupled with high actionability; previous studies confirm that these kinase-domain alterations exhibit pronounced sensitivity to *HER2*-targeted therapies [32]. Meanwhile, patients in the VUS category showed intermediate outcomes, but because VUS represents a heterogeneous category with uncertain functional significance, these observations should be interpreted descriptively rather than as evidence of a biologically homogeneous prognostic subgroup. Furthermore, our analysis suggests that *HER2* gene-altered NSCLC, regardless of the specific subtype or protein expression level, tends to be an immunologically “cold” phenotype with low PD-L1 expression. This finding, consistent with prior studies [33–36], may help explain the limited efficacy of immune checkpoint inhibitor (ICI) monotherapy in this population. Consequently, therapeutic strategies could consider *HER2*-directed agents, such as ADCs, or combination regimens designed to overcome this immune-excluded microenvironment [37], rather than relying solely on ICIs.

This study has several limitations. First, this single-center retrospective study included patients with surgically resected NSCLC and therefore predominantly represents resectable disease. Accordingly, the findings may not be generalizable to patients with advanced or unresectable *HER2*-altered NSCLC. Validation in larger cohorts that include patients with advanced-stage disease is warranted. Second, the *HER2* amplification subgroup was relatively small (*n* = 28), which limited statistical power and increased the susceptibility of the survival analyses to residual confounding by pathological stage and other clinicopathological factors. Although *HER2* amplification was associated with inferior DFS and OS in Kaplan–Meier analyses and with shorter DFS in univariable Cox analysis, it was not retained as an independent prognostic factor in the multivariable Cox model and was likewise not independently associated with DFS in the additional treatment-adjusted analysis. Therefore, the survival findings related to *HER2* amplification should be considered exploratory and interpreted cautiously. Third, *HER2* amplification was assessed using NGS-based copy-number analysis without orthogonal confirmation. *HER2* FISH was not performed in any of the cases with NGS-detected *ERBB2* copy-number amplification in this retrospective cohort; therefore, concordance between the NGS-based amplification calls and FISH could not be evaluated. Moreover, NGS-based copy-number estimation may be influenced by tumor purity, ploidy, overall copy-number background, sequencing depth, and platform-specific analytical criteria. Fourth, HER2 IHC results were available only for a subset of patients because HER2 IHC testing was not universally performed during routine clinical management. This may have introduced selection bias and limited the interpretation of associations between HER2 protein expression, molecular alterations, and clinical outcomes. Fifth, the *HER2* VUS category comprises a heterogeneous group of variants with uncertain functional significance. Therefore, findings related to this category should be considered descriptive and exploratory and require further functional and clinical validation. Finally, although available treatment information was retrospectively collected, postoperative and post-recurrence treatment information remained incomplete for some patients. Postoperative treatment differed among *HER2* alteration subgroups, and treatment selection bias or confounding by indication could not be fully excluded when interpreting survival outcomes. Among patients with available treatment information, no *HER2*-targeted therapy was documented in the adjuvant setting, whereas one patient received trastuzumab deruxtecan after recurrence; therefore, the impact of post-recurrence treatment on OS could not be fully assessed. Future prospective studies with larger, treatment-annotated cohorts, systematic HER2 IHC assessment, orthogonal validation of NGS-detected copy-number alterations, and integrated molecular profiling are warranted to validate these findings.

## Conclusion

This study demonstrates heterogeneous clinicopathological, genomic, protein expression, and survival profiles of *HER2* alteration categories in surgically resected NSCLC. *HER2* amplification was associated with adverse clinicopathological characteristics, higher frequencies of concurrent *TP53* and *EGFR* alterations, and higher HER2 protein expression. Although *HER2* amplification was associated with inferior survival in Kaplan–Meier and univariable analyses, it was not retained as an independent prognostic factor after adjustment for clinicopathological variables and postoperative adjuvant treatment. The relatively favorable survival outcomes observed for Y772_A775dup and other TKD mutations should be considered exploratory. These findings support alteration-specific interpretation of *HER2* results and warrant validation in larger, independent, and treatment-annotated cohorts.

## Supplementary Information

Supplementary Material 1.

## Data Availability

Some or all of the data used or generated in this study are available upon request from the corresponding author.
