# Supplementary material for: Clinicopathologic and prognostic value of HER2 alterations in non-small cell lung cancer
Source: Respir Res. 2026 Aug 10;27:434. doi: 10.1186/s12931-026-03864-3 (PMC13737758; doi:10.1186/s12931-026-03864-3)
Supplement: Supplementary file 1 [file 12931_2026_3864_MOESM1_ESM.docx]

Clinicopathologic and Prognostic Value of *HER2* Alterations in Non-Small Cell Lung Cancer

Yicong Lin^1,2,3,4,#^, Qianqian Xue^1,2,3,4,#^, Bingxia Zhang^1,2,3^, Yue Wang^1,2,3^, Qiang Zheng^1,2,3^, Yan Jin^1,2,3,✉^, Yuan Li^1,2,3,✉^

^1^Department of Pathology, Fudan University Shanghai Cancer Center, Shanghai, China.

^2^Department of Oncology, Shanghai Medical College, Fudan University, Shanghai, China.

^3^Institute of Pathology, Fudan University, Shanghai, China.

^4^Department of Thoracic Surgery, Fudan University Shanghai Cancer Center, Shanghai, China.

^✉^Corresponding author.

^#^Contributed equally.

Supplementary Table 1. Detailed distribution of pT stage, pN stage, and pathological stage in patients with *HER2* gene-altered NSCLC

|  | Number of patients (%) with *HER2* alteration | | | |
| --- | --- | --- | --- | --- |
|  | Mutation | | Amplification |  |
| Variables | Pathogenic mut (n=230) | VUS (n=104) | Amp (n=28) | p value |
| pT stage |  |  |  | <0.0001 |
| T1 | 207(90.00) | 78(75.00) | 11(39.29) |  |
| T2 | 16(6.96) | 17(16.35) | 12(42.86) |  |
| T3 | 5(2.17) | 7(6.73) | 3(10.71) |  |
| T4 | 2(0.87) | 2(1.92) | 2(7.14) |  |
| pN stage |  |  |  | 0.0199 |
| N0 | 199(86.52) | 91(87.50) | 19(67.86) |  |
| N1 | 10(4.35) | 8(7.69) | 5(17.86) |  |
| N2 | 21(9.13) | 5(4.81) | 4(14.28) |  |
| N3 | 0(0.00) | 0(0.00) | 0(0.00) |  |
| pStage |  |  |  | <0.0001 |
| I | 194(84.35) | 83(79.81) | 11(39.29) |  |
| II | 11(4.78) | 12(11.54) | 6(21.43) |  |
| III | 23(10.00) | 9(8.65) | 8(28.57) |  |
| IV | 2(0.87) | 0(0.00) | 3(10.71) |  |

pT stage, pathologic T stage; pN stage, pathologic N stage; pStage, pathological stage; *HER2*, Human epidermal growth factor receptor 2.

Supplementary Table 2. Clinicopathologic characteristics of specific *HER2* gene mutation variants in NSCLC

|  | Number of patients (%) with *HER2* alteration | | | | | | | | | |
| --- | --- | --- | --- | --- | --- | --- | --- | --- | --- | --- |
| Clinicopathological variable | Y772_A775dup(n=141) | G776delinsVC(n=19) | S310F(n=13) | V659E(n=8) | G778_P780dup(n=6) | Amp+Mut (n=4) | Other pathogenic mut(n=39) | VUS (n=104) | Amp (n=28) | p value |
| Age |  |  |  |  |  |  |  |  |  | <0.0001 |
| ≤59 years | 108(76.60) | 14(73.68) | 5(38.46) | 6(75.00) | 3(50.00) | 2(50.00) | 27(69.23) | 36(34.62) | 7(25.00) |  |
| >59 years | 33(23.40) | 5(26.32) | 8(61.54) | 2(25.00) | 3(50.00) | 2(50.00) | 12(30.77) | 68(65.38) | 21(75.00) |  |
| Sex |  |  |  |  |  |  |  |  |  | <0.0001 |
| Female | 97(68.79) | 12(63.16) | 7(53.85) | 8(100.00) | 4(66.67) | 2(50.00) | 28(71.79) | 44(42.31) | 10(35.71) |  |
| Male | 44(31.21) | 7(36.84) | 6(46.15) | 0(0.00) | 2(33.33) | 2(50.00) | 11(28.21) | 60(57.69) | 18(64.29) |  |
| Smoking |  |  |  |  |  |  |  |  |  | <0.0001 |
| Never | 119(84.40) | 16(84.21) | 8(61.54) | 7(87.50) | 6(100.00) | 2(50.00) | 33(84.62) | 58(55.77) | 12(42.86) |  |
| Ever | 22(15.60) | 3(15.79) | 5(38.46) | 1(12.50) | 0(0.00) | 2(50.00) | 6(15.38) | 46(44.23) | 16(57.14) |  |
| Histology |  |  |  |  |  |  |  |  |  | <0.0001 |
| LUAD | 141(100.00) | 19(100.00) | 13(100.00) | 8(100.00) | 5(83.33) | 4(100.00) | 38(97.44) | 86(82.69) | 21(75.00) |  |
| LUSC | 0(0.00) | 0(0.00) | 0(0.00) | 0(0.00) | 0(0.00) | 0(0.00) | 0(0.00) | 9(8.65) | 4(14.29) |  |
| Other | 0(0.00) | 0(0.00) | 0(0.00) | 0(0.00) | 1(16.67) | 0(0.00) | 1(2.56) | 9(8.65) | 3(10.71) |  |
| Differentiation |  |  |  |  |  |  |  |  |  | <0.0001 |
| Well-differentiated | 30(21.28) | 3(15.79) | 0(0.00) | 0(0.00) | 0(0.00) | 0(0.00) | 4(10.26) | 5(4.81) | 0(0.00) |  |
| Moderately-differentiated | 32(22.70) | 7(36.84) | 12(92.31) | 2(25.00) | 3(50.00) | 0(0.00) | 13(33.33) | 40(38.46) | 2(7.14) |  |
| Poorly-differentiated | 26(18.44) | 4(21.05) | 0(0.00) | 3(37.50) | 2(33.33) | 4(100.00) | 12(30.77) | 48(46.15) | 25(89.29) |  |
| NA | 53(37.59) | 5(26.32) | 1(7.69) | 3(37.50) | 1(16.67) | 0(0.00) | 10(25.64) | 11(10.58) | 1(3.57) |  |
| pT stage |  |  |  |  |  |  |  |  |  | 0.032 |
| T1-T2 | 137(97.16) | 19(100.00) | 13(100.00) | 8(100.00) | 6(100.00) | 3(75.00) | 37(94.87) | 95(91.35) | 23(82.14) |  |
| T3-T4 | 4(2.84) | 0(0.00) | 0(0.00) | 0(0.00) | 0(0.00) | 1(25.00) | 2(5.13) | 9(8.65) | 5(17.86) |  |
| pN stage |  |  |  |  |  |  |  |  |  | 0.1357 |
| N0 | 122(86.52) | 17(89.47) | 13(100.00) | 7(87.50) | 6(100.00) | 3(75.00) | 31(79.49) | 91(87.50) | 19(67.86) |  |
| N1-2 | 19(13.48) | 2(10.53) | 0(0.00) | 1(12.50) | 0(0.00) | 1(25.00) | 8(20.51) | 13(12.50) | 9(32.14) |  |
| pStage |  |  |  |  |  |  |  |  |  | 0.0029 |
| I-II | 125(88.65) | 17(89.47) | 13(100.00) | 7(87.50) | 6(100.00) | 3(75.00) | 34(87.18) | 95(91.35) | 17(60.71) |  |
| III-IV | 16(11.35) | 2(10.53) | 0(0.00) | 1(12.50) | 0(0.00) | 1(25.00) | 5(12.82) | 9(8.65) | 11(39.29) |  |
| Vascular invasion |  |  |  |  |  |  |  |  |  | <0.0001 |
| Absent | 114(80.85) | 17(89.47) | 8(61.54) | 6(75.00) | 4(66.67) | 2(50.00) | 30(76.92) | 76(73.08) | 10(35.71) |  |
| Present | 22(15.60) | 2(10.53) | 1(7.69) | 2(25.00) | 1(16.67) | 1(25.00) | 8(20.51) | 22(21.15) | 16(57.14) |  |
| NA | 5(3.55) | 0(0.00) | 4(30.77) | 0(0.00) | 1(16.67) | 1(25.00) | 1(2.56) | 6(5.77) | 2(7.14) |  |
| Perineural invasion |  |  |  |  |  |  |  |  |  | 0.2055 |
| Absent | 138(97.87) | 19(100.00) | 13(100.00) | 7(87.50) | 6(100.00) | 3(75.00) | 38(97.44) | 98(94.23) | 25(89.29) |  |
| Present | 2(1.42) | 0(0.00) | 0(0.00) | 1(12.50) | 0(0.00) | 1(25.00) | 0(0.00) | 3(2.88) | 2(7.14) |  |
| NA | 1(0.71) | 0(0.00) | 0(0.00) | 0(0.00) | 0(0.00) | 0(0.00) | 1(2.56) | 3(2.88) | 1(3.57) |  |
| Pleural invasion |  |  |  |  |  |  |  |  |  | 0.3044 |
| Absent | 124(87.94) | 15(78.95) | 12(92.31) | 6(75.00) | 5(83.33) | 2(50.00) | 30(76.92) | 87(83.65) | 19(67.86) |  |
| Present | 11(7.80) | 2(10.53) | 0(0.00) | 2(25.00) | 1(16.67) | 1(25.00) | 5(12.82) | 12(11.54) | 7(25.00) |  |
| NA | 6(4.26) | 2(10.53) | 1(7.69) | 0(0.00) | 0(0.00) | 1(25.00) | 4(10.26) | 5(4.81) | 2(7.14) |  |

*HER2*, Human epidermal growth factor receptor 2; Pathogenic mut, Pathogenic mutation; VUS, Variants of uncertain significance; Amp, Amplification; pT stage, pathologic T stage; pN stage, pathologic N stage; pStage, pathological stage; NA, Not available.

Supplementary Table 3.1. Postoperative treatment characteristics according to *HER2* alteration category.

| Treatments | Total (n=362) | Pathogenic mutation (n=230) | Amplification (n=28) | VUS(n=104) |
| --- | --- | --- | --- | --- |
| Postoperative treatment information available | 310/362 (85.6%) | 199/230 (86.5%) | 27/28 (96.4%) | 84/104 (80.8%) |
| Postoperative treatment information unknown | 52/362 (14.4%) | 31/230 (13.5%) | 1/28 (3.6%) | 20/104 (19.2%) |
| Postoperative adjuvant treatment among available cases | 72/310 (23.2%) | 32/199 (16.1%) | 19/27 (70.4%) | 21/84 (25.0%) |
| Adjuvant chemotherapy | 62/310 (20.0%) | 31/199 (15.6%) | 16/27 (59.3%) | 15/84 (17.9%) |
| Adjuvant targeted therapy | 11/310 (3.5%) | 0/199 (0.0%) | 4/27 (14.8%) | 7/84 (8.3%) |
| Adjuvant radiotherapy | 6/310 (1.9%) | 4/199 (2.0%) | 1/27 (3.7%) | 1/84 (1.2%) |
| Documented adjuvant *HER2*-targeted therapy among available cases | 0/310 (0.0%) | 0/199 (0.0%) | 0/27 (0.0%) | 0/84 (0.0%) |
| No postoperative adjuvant treatment among available cases | 238/310 (76.8%) | 167/199 (83.9%) | 8/27 (29.6%) | 63/84 (75.0%) |

Values are presented as n/N (%). Postoperative treatment variables were summarized among patients with available postoperative treatment information. Cases with insufficient documentation were coded as unknown rather than untreated. Treatment categories were not mutually exclusive. VUS, Variants of uncertain significance.

Supplementary Table 3.2. Post-recurrence treatment according to *HER2* alteration category.

| Treatments | Total (n=362) | Pathogenic mutation (n=230) | Amplification (n=28) | VUS(n=104) |
| --- | --- | --- | --- | --- |
| DFS event (recurrence or death) | 84/362 (23.2%) | 42/230 (18.3%) | 16/28 (57.1%) | 26/104 (25.0%) |
| Documented recurrence or metastasis | 66/362 (18.2%) | 34/230 (14.8%) | 14/28 (50.0%) | 18/104 (17.3%) |
| Death without documented recurrence or metastasis | 18/362 (5.0%) | 8/230 (3.5%) | 2/28 (7.1%) | 8/104 (7.7%) |
| Post-recurrence treatment information available | 43/66 (65.2%) | 22/34 (64.7%) | 8/14 (57.1%) | 13/18 (72.2%) |
| Post-recurrence treatment information unknown | 23/66 (34.8%) | 12/34 (35.3%) | 6/14 (42.9%) | 5/18 (27.8%) |
| Post-recurrence treatment among available cases | 37/43 (86.0%) | 19/22 (86.4%) | 7/8 (87.5%) | 11/13 (84.6%) |
| No post-recurrence treatment among available cases | 6/43 (14.0%) | 3/22 (13.6%) | 1/8 (12.5%) | 2/13 (15.4%) |
| Post-recurrence chemotherapy | 25/43 (58.1%) | 17/22 (77.3%) | 4/8 (50.0%) | 4/13 (30.8%) |
| Post-recurrence targeted therapy | 14/43 (32.6%) | 4/22 (18.2%) | 2/8 (25.0%) | 8/13 (61.5%) |
| Post-recurrence radiotherapy  Post-recurrence immunotherapy  Documented post-recurrence *HER2*-targeted therapy | 9/43 (20.9%)  21/43 (48.8%)  1/43 (2.3%) | 6/22 (27.3%)  14/22 (63.6%)  1/22 (4.5%) | 3/8 (37.5%)  5/8 (62.5%)  0/8 (0.0%) | 0/13 (0.0%)  2/13 (15.4%)  0/13 (0.0%) |

Values are presented as n/N (%). Post-recurrence treatment analyses were restricted to patients with documented recurrence or metastasis; patients who died without documented recurrence or metastasis were excluded. Cases with insufficient documentation were coded as unknown rather than untreated. Treatment modalities were summarized among patients with available post-recurrence treatment information and were not mutually exclusive. One patient in the pathogenic mutation group received three cycles of trastuzumab deruxtecan (T-DXd; DS-8201) after recurrence. VUS, variants of uncertain significance.

Supplementary Table 3.3. Treatment-adjusted Cox regression analysis for disease-free survival

| **Covariate** | **Treatment-adjusted model** | | **Sensitivity model** | |
| --- | --- | --- | --- | --- |
|  | **HR (95% CI)** | **P value** | **HR (95% CI)** | **P value** |
| Age >59 years | 2.27 (1.43–3.60) | <0.001 | 2.67 (1.60–4.44) | <0.001 |
| pStage II | 1.41 (0.67–2.97) | 0.366 | 1.02 (0.44–2.36) | 0.970 |
| pStage III | 1.74 (0.85–3.55) | 0.127 | 1.46 (0.66–3.26) | 0.350 |
| pStage IV | 4.20 (1.23–14.40) | 0.022 | 3.35 (0.97–11.64) | 0.056 |
| *HER2* amplification | 1.17 (0.62–2.22) | 0.631 | 1.27 (0.66–2.45) | 0.476 |
| Postoperative adjuvant treatment: yes | 3.57 (1.77–7.18) | <0.001 | 4.20 (2.02–8.73) | <0.001 |
| Postoperative adjuvant treatment: unknown | 1.59 (0.78–3.24) | 0.200 | — | — |

The treatment-adjusted model included age, pStage, *HER2* amplification status, and postoperative adjuvant treatment, with unknown treatment status retained as a separate category. The sensitivity model was restricted to patients with available postoperative treatment information. Patients with missing data for other model covariates were excluded from the respective analyses. Reference categories were age ≤59 years, pStage I, *HER2* non-amplification, and no postoperative adjuvant treatment. For modeling purposes, the *HER2* non-amplification category included pathogenic mutations and VUS and does not imply biological homogeneity between these categories. pStage, pathological stage; HR, hazard ratio; CI, confidence interval; DFS, disease-free survival.
